# Molecular Analysis of Antibiotic Resistance Determinants and Plasmids in Malaysian Isolates of Multidrug Resistant *Klebsiella pneumoniae*

**DOI:** 10.1371/journal.pone.0133654

**Published:** 2015-07-23

**Authors:** Farah Al-Marzooq, Mohd Yasim Mohd Yusof, Sun Tee Tay

**Affiliations:** Department of Medical Microbiology, Faculty of Medicine, University of Malaya, Kuala Lumpur, Malaysia; University of Minnesota, UNITED STATES

## Abstract

Infections caused by multidrug resistant *Klebsiella pneumoniae* have been increasingly reported in many parts of the world. A total of 93 Malaysian multidrug resistant *K*. *pneumoniae* isolated from patients attending to University of Malaya Medical Center, Kuala Lumpur, Malaysia from 2010-2012 were investigated for antibiotic resistance determinants including extended-spectrum beta-lactamases (ESBLs), aminoglycoside and trimethoprim/sulfamethoxazole resistance genes and plasmid replicons. CTX-M-15 (91.3%) was the predominant ESBL gene detected in this study. *aacC2* gene (67.7%) was the most common gene detected in aminoglycoside-resistant isolates. Trimethoprim/sulfamethoxazole resistance (90.3%) was attributed to the presence of *sul1* (53.8%) and *dfrA* (59.1%) genes in the isolates. Multiple plasmid replicons (1-4) were detected in 95.7% of the isolates. FIIK was the dominant replicon detected together with 13 other types of plasmid replicons. Conjugative plasmids (1-3 plasmids of ~3-100 kb) were obtained from 27 of 43 *K*. *pneumoniae* isolates. An ESBL gene (either CTX-M-15, CTX-M-3 or SHV-12) was detected from each transconjugant. Co-detection with at least one of other antibiotic resistance determinants [*sul1*, *dfrA*, *aacC2*, *aac(6ˊ)-Ib*, *aac(6ˊ)-Ib-cr* and *qnrB*] was noted in most conjugative plasmids. The transconjugants were resistant to multiple antibiotics including β-lactams, gentamicin and cotrimoxazole, but not ciprofloxacin. This is the first study describing the characterization of plasmids circulating in Malaysian multidrug resistant *K*. *pneumoniae* isolates. The results of this study suggest the diffusion of highly diverse plasmids with multiple antibiotic resistance determinants among the Malaysian isolates. Effective infection control measures and antibiotic stewardship programs should be adopted to limit the spread of the multidrug resistant bacteria in healthcare settings.

## Introduction

*Klebsiella pneumoniae* is a major cause of community and healthcare associated infections [1]. Infections caused by multidrug resistant *K*. *pneumoniae*, have been increasingly reported in many clinical settings [1–3]. Besides extended-spectrum β-lactamase (ESBL) production, *K*. *pneumoniae* is frequently known to be resistant to multiple antimicrobial agents including fluoroquinolones, aminoglycosides and trimethoprim/sulfamethoxazole [4]. These infections are usually associated with high morbidity and mortality, long hospital stay and high healthcare costs [4,5].

Horizontal transfer of antibiotic resistance genes has been considered as one of the most important mechanisms for the dissemination of multidrug resistance among bacteria [6]. The evolution and dissemination of resistance genes occur mostly through the transmission of plasmids which are highly diverse with respect to size, modes of replication and transcription, host ranges and genes that they carry [7, 8]. IncF plasmids are one of the most common plasmid types which are usually associated with the spread of antimicrobial resistance determinants in *Enterobacteriaceae* [9]. FIIK is a common plasmid replicon in *Klebsiella* species [10]. Recently, FIIK plasmids with multiple antibiotic resistance genes including CTX-M-15 have been identified in *K*. *pneumoniae* [11].

This study was conducted to investigate antibiotic resistance determinants for extended-spectrum beta-lactamases (ESBLs), aminoglycosides and trimethoprim/sulfamethoxazole in 93 Malaysian *K*. *pneumoniae* isolates. This study also aimed to identify plasmid replicons in the isolates and to test the transmissibility of plasmids carrying ESBL genes by conjugation in a subset of these isolates.

## Materials and Methods

### Bacterial isolates

A group of 93 non-duplicated Malaysian multidrug resistant *K*. *pneumoniae* isolates investigated previously for their ciprofloxacin resistance mechanisms were used in this study [12]. Full characterization of the antibiotic resistance genes carried by two of these isolates (strain K24 and strain NDM-2012) had been reported previously [13, 14].

### Antimicrobial susceptibility testing

Antimicrobial susceptibilities were determined by disk diffusion and E-test method in accordance to the Clinical and Laboratory Standards Institute (CLSI) guidelines [15]. The following antibiotic disks (Oxoid Ltd, Basingstoke, Hampshire, UK) were used: ampicillin (10 μg), ampicillin-sulbactam (10/10 μg), amoxicillin/clavulanate (20/10 μg), ertapenem (10 μg), imipenem (10 μg), meropenem (10 μg), cefoxitin (30 μg), ceftriaxone (30 μg), cefuroxime (30 μg) and cefoperazone (30 μg). E-test strips (BioMerieux, Marcy l'Etoile, France) were used to define the minimum inhibitory concentrations (MICs) for aztreonam, ceftazidime, cefotaxime, piperacillin-tazobactam, gentamicin, amikacin and trimethoprim/sulfamethoxazole. ESBL production by each isolate was detected by cefpodoxime combination disk kit (Oxoid Ltd, Basingstoke, Hampshire, UK) and cefepime/cefepime + clavulanic acid E—test strips (BioMerieux, Marcy l'Etoile, France).

### Molecular identification of antibiotic resistance determinants

DNA was extracted from fresh bacterial colonies using a QIAamp DNA minikit (Qiagen, Germany). Amplification of all targets was performed using 5 × HOT FIREPol Blend Master Mix (Solis BioDyne, Estonia) on a Veriti 96 well Thermal Cycler (Applied Biosystems, USA).

ß-lactamase genes were detected by several PCR assays targeting CTX-M groups (1, 2, 8, 9 & 25), SHV, TEM, carbapenemase genes (IMP, VIM, KPC and NDM), AmpC genes (ACC, FOX, MOX, DHA, CIT and EBC) and minor ESBL genes (VEB, GES and PER) [14]. The isolates were also screened for various OXA groups including OXA-48 [14], OXA-1 group (-1, -30, -31 and -47), OXA-2 group (-2, -3, -15, -21, -32, -34, -36, -46, -53, -141, -144, -161-118, and -119), OXA-51 group (-51, -64 to -71, -75 to -80, -82 to -84, -86 to -95, -98 to -100, -106 to -113, -115 to -117, -128, -130 to -132, -138, -144, -148 to -150, and -172 to -180), OXA-5 and OXA-10 group (OXA-4, -7, -10, -11, -13, -14, -16, -17, -19, -28, -56, -74, -101, -129, -142, -145 & -147), and OXA-58 group (-58, -96, -97, -164) [16].

PCR was also used to detect the presence of the genes encoding acetyltransferases (*aacC1* and *aacC2*), nucleotidyltransferase (*aadB*), phosphotransferase (*aphA6*), 16S rRNA methylases (*armA* and *rmtB*), *sul* and *dfrA* genes [14]. To confirm the PCR results, randomly selected amplicons were purified and sequenced. The nucleotides and deduced protein sequences were analyzed with BLAST search engine (http://blast.ncbi.nlm.nih.gov/Blast.cgi) and BioEdit software (version 7.1.3.0).

### PCR-based plasmid replicon typing (PBRT) method

The resistance plasmids of the 93 isolates were characterized using a commercial kit (Diatheva, Italy). Total bacterial DNA was prepared and used as a PCR template in accordance to the PBRT kit manufacturer’s protocol (http://www.diatheva.com/catalogue/pbrt/pbrt-kit-pcr-based-replicon-typing-details). Eight multiplex PCR assays were used for amplification of 25 replicons (incompatibility groups): HI1, HI2, I1, I2, X1, X2, L/M, N, FIA, FIB, FIC, FII, FIIS, FIIK, W, Y, P, A/C, T, K, U, R, B/O, HIB-M and FIB-M, which are representative of the major plasmid incompatibility groups and replicase genes on resistance plasmids circulating among *Enterobacteriaceae* [17]. Sequence typing of IncF replicons (FIIK, FII, FIA and FIB) was conducted according to the plasmid MLST protocol (http://pubmlst.org/plasmid/). IncF replicon sequences were analyzed using plasmid MLST website (http://pubmlst.org/plasmid/). FAB formulae was determined by using allele type and number for each replicon [10].

### Conjugation

Conjugation was carried out by broth mating (at a ratio of 1:4) in order to test the transmissibility of ESBL plasmid from *K*. *pneumoniae* isolates as donors to a recipient *Escherichia coli* strain J53 AzR (resistant to azide) [18]. A total of 43 *K*. *pneumoniae* isolates were selected based on types of ESBL genes and plasmid replicons. *E*. *coli* clones carrying ESBL plasmid (transferred from the donor) were selected on Luria-Bertani agar plates containing azide (100 μg/ml) and cefotaxime (2 μg/ml).

#### Characterization of the transconjugants

For detection of beta-lactamase production by transconjugants, nitrocefin kit (Oxoid, UK) was used as recommended by the manufacturer. Phenotypic detection of ESBL production by the transconjugants was performed using Oxoid combination disk method (cefpodoxime and cefpodoxime with clavulanate). Antimicrobial susceptibilities of the transconjugants were determined by disk diffusion and E-test methods. Susceptibility profiles and MICs of the transconjugants were compared to those of the donors and *E*. *coli* (J53 AzR) recipient strain. All the transconjugants were screened using PCR for the plasmid replicons and antibiotic resistance genes in their donors. Representative amplicons were sequenced for the purpose of confirmation.

Plasmid DNA was extracted from the donors and transconjugants using a plasmid midi kit (Qiagen, Germany). To determine the plasmid size and number, extracted plasmids from donors and transconjugants were separated on a 0.8% agarose gel prestained with 0.5 μg/ml ethidium bromide (Thermo Scientific, US). Supercoiled DNA ladder (New England Biolabs, UK) was used as a molecular weight standard for the plasmid size estimation. Electrophoresis was performed in 0.5X TBE buffer at 80 V for 3 hr.

Transconjugants plasmids were digested with the restriction enzyme *EcoRI*-HF (New England Biolabs, UK) [19]. Restriction fragments were separated on a 0.8% agarose gel prestained with 0.5 μg/ml ethidium bromide (Thermo Scientific, US) at 80 V for 3 hrs and visualized following gel electrophoresis. Lambda DNA/HindIII Marker, Ready-to-Use (Thermo Fisher Scientific, Lithuania) was used as a molecular weight standard. Plasmid restriction profiles were compared using Bionumerics software, version 7.0 (Applied Maths, Kortrijk, Belgium). Cluster analysis was carried out by the unweighted pair group method with arithmetic mean (UPGMA) algorithm by defining a similarity (Dice) coefficient. Cluster designation was based on plasmid profiles with ≥ 80% relatedness.

### Statistical analyses

Antibiotic resistance rates were expressed as percentages of the total number of isolates. All the statistical tests were performed using PASW Statistics version 18 (SPSS Inc., Chicago, IL, US). A p-value <0.05 was considered statistically significant. Paired-sample t-test was used to compare the MICs of transconjugants to their donors (*K*. *pneumoniae*) and recipients (*E*. *coli* strain J53 AzR), respectively.

## Results

### Antibiotic susceptibilities of the *K*. *pneumoniae* isolates

Both cefpodoxime combination disk kit (Oxoid Ltd, Basingstoke, Hampshire, UK) and cefepime/ cefepime + clavulanic acid E—test strips (BioMerieux, Marcy l'Etoile, France) confirmed that all *K*. *pneumoniae* isolates were ESBL producers. The isolates demonstrated high resistance rates against ampicillin (100%), aztreonam (98.9%) and cephalosporins including ceftriaxone (100%), cefoperazone (100%), cefotaxime (100%), cefuroxime (98.9%) and ceftazidime (97.8%). Most of the isolates were susceptible to cefoxitin, except three (3.2%) isolates (strain K106, M40 and NDM-2012). The resistance rate against piperacillin-tazobactam (43%) was lower than the rates of other β-lactam/β-lactamase inhibitor combinations (94.6% and 98.9% for amoxicillin-clavulanate and ampicillin-sulbactam, respectively). Non-susceptibility to trimethoprim/sulfamethoxazole, gentamicin and amikacin was observed in 90.3%, 74% and 5.4% of the isolates, respectively. Non-susceptibility to ciprofloxacin was noted in 71% of the isolates [12]. 92 isolates were susceptible to carbapenems (imipenem, meropenem, and ertapenem). Strain NDM-2012 demonstrated resistance to all antibiotics tested including carbapenems [14]. Table 1 shows MIC ranges, MIC_50_ and MIC_90_ for the 93 multidrug resistant *K*. *pneumoniae* isolates investigated in this study.

**Table 1:** MICs ranges, MIC_50_ and MIC_90_ for the 93 multidrug resistant *K*. *pneumoniae* isolates investigated in this study.

**Antibiotic**	**MIC (μg/ml)**		
	range	MIC_50_	MIC_90_
Ceftazidime	2–≥256	24	≥256
Cefotaxime	4–≥256	≥256	≥256
Aztreonam	4–≥256	48	≥256
Piperacillin-tazobactam	2–≥128	16	≥128
Gentamicin	0.19–≥256	24	96
Amikacin	0.5–≥256	4	16
Ciprofloxacin	0.032–≥32	2	≥32
Trimethoprim/sulfamethoxazole	0.125–≥32	≥32	≥32

### Antibiotic resistance determinants in the *K*. *pneumoniae* isolates

CTX-M-15 (91.3%, n = 85) was the most prevalent ESBL gene detected in this study. Other CTX-M types including CTX-M-3 and CTX-M-63 were detected at low rates (1.1%, n = 1 each). SHV gene was detected in 78.5% (n = 73) of the isolates; however, SHV-12 (6.5%, n = 6) was the only ESBL-SHV type detected in this study. Other SHV types (SHV-1, -11, -27, -28, -75, and -83) detected in this study were non-ESBLs (http://www.lahey.org/Studies/). A novel SHV gene (SHV-144) was detected in one isolate (strain K24) [13]. OKP (Other *K*. *pneumoniae* β-lactamases) was amplified from two isolates using SHV screening primers [20, 21]. Other β-lactamase genes detected in this study were TEM (63.4%, n = 59) and OXA-1 like (34.4%, n = 32). DHA-1 gene (2.2%, n = 2) was the only AmpC beta-lactamase gene detected from two cefoxitin-resistant isolates (strain K106 and M40). Carbapenemase genes (IMP, VIM, KPC, OXA-48 like and NDM) were not detected from 92 isolates susceptible to carbapenems. Both NDM-1 and OXA-232 genes were amplified from the carbapenem-resistant strain NDM-2012 [14].

*aacC2* (n = 63; 67.7%) was detected in most of gentamicin non-susceptible isolates (n = 69, 74%); thus, the association between the presence of *aacC2* gene and non-susceptibility to gentamicin was statistically significant (p<0.05). Other less common aminoglycoside resistance genes detected in this study were *aadB* gene which was detected in a gentamicin-resistant isolate (1.1%), and *armA* gene (1.1%) which was amplified from the carbapenem-resistant strain NDM-2012 [14]. Neither 16S rRNA methylase (*rmtB*) nor phosphotransferase gene (*aphA6*) was detected in this study. Trimethoprim/sulfamethoxazole resistance genes including *sul1* and *dfrA* were detected in 50 (53.8%) and 55 (59.1%) isolates, respectively.

### Plasmid replicon typing in *K*. *pneumoniae* isolates

A total of 14 plasmid replicon types were detected from 95.7% (n = 89) of the isolates. Except four isolates, 1–4 replicons were detected from each *K*. *pneumoniae* isolate investigated in this study. Table 2 demonstrates the number and type of plasmid replicons detected in this study, and the antibiotic resistance genes detected in each isolate. FIIK was the most prevalent replicon identified in 84 (90.3%) isolates. It was detected either alone in 50 (53.8%) isolates or accompanied by 1–3 other replicons in 34 (36.5%) isolates. Other plasmid replicons detected were R (20.4%), FIB-M (7.6%), N (5.4%), HI2 (5.4%), HIB-M (3.2%), Y (3.2%), FII (3.2%), FIA (2.2%), FIB (2.2%), I1 (2.2%), A/C (1.1%), X1 (1.1%) and K (1.1%). The full list of plasmid replicons and antibiotic resistance genes detected in each *K*. *pneumoniae* isolate is shown in S1 Table.

**Table 2:** Plasmid replicons and antibiotic resistance genes detected in 93 *K*. *pneumoniae* isolates.

**No. of replicons**			Antibiotic resistance genes (detection rates)																	
			Beta-lactamases									PMQR			Aminoglycosides				Cotrimoxazole	
	Plasmid replicons	No. (%) of isolates	CTX-M-15 (91.3%)	CTX-M-3 (1.1%)	CTX-M-63 (1.1%)	SHV-12 (6.5%)	Other SHV types (72%)	TEM (63.4%)	OXA-1 (34.4%)	OXA-232 + NDM-1 (1.1%)	DHA-1 (2.2%)	*qnrB* (59.1%)	*qnrS* (1.1%)	*aac(6’)-Ib-cr* (65.6%)	*aacC2* (67.7%)	*aadB* (1.1%)	*armA* (1.1%)	*aac(6’)-Ib* (14%)	*sul1* (53.8%)	*dfrA* (59.1%)
0	Non-typable	4 (4.3)	**+**	‒	‒	‒	**V**	**+**	**V**	‒	**‒**	**V**	‒	**V**	**V**	‒	‒	‒	‒	**+**
1	**FIIK**	50 (53.8)	**+**	‒	‒	‒	**V**	**V**	**V**	‒	**V**	**V**	‒	**V**	**V**	‒	‒	**V**	**V**	**V**
	FIB-M	2 (2.2)	**+**	‒	‒	‒	**+**	**+**	**+**	‒	‒	**+**	‒	**+**	**+**	‒	‒	‒	**V**	**+**
	N	1 (1.1)	**‒**	**+**	‒	‒	**+**	‒	‒	‒	‒	‒	‒	‒	‒	‒	‒	‒	**+**	**‒**
2	**FIIK**, R	14 (15.1)	**+**	‒	‒	‒	**V**	**V**	**V**	‒	‒	**V**	‒	**V**	**V**	‒	‒	**V**	**V**	**V**
	**FIIK**, FIB-M	3 (3.2)	**+**	‒	‒	‒	**V**	**V**	**V**	‒	‒	**V**	‒	**+**	**V**	‒	‒	‒	**+**	**V**
	**FIIK**, FIA	1 (1.1)	**+**	‒	‒	‒	**+**	**+**	**+**	‒	‒	**+**	‒	**+**	**+**	‒	‒	‒	**+**	**+**
	**FIIK**, FII	1 (1.1)	**+**	‒	‒	‒	**+**	**+**	‒	‒	‒	**+**	‒	**+**	**+**	‒	‒	‒	**+**	‒
	**FIIK**, HIB-M	1 (1.1)	**+**	‒	‒	‒	**+**	**+**	‒	‒	‒	‒	‒	**+**	**‒**	‒	‒	‒	**+**	‒
	**FIIK**, I1	1 (1.1)	**+**	‒	‒	‒	**+**	**+**	‒	‒	‒	‒	‒	‒	**‒**	‒	‒	‒	‒	**+**
	**FIIK**, K	1 (1.1)	**+**	‒	‒	‒	**+**	**‒**	‒	‒	‒	‒	‒	‒	**+**	‒	‒	**+**	‒	**‒**
	**FIIK**, N	1 (1.1)	**+**	‒	‒	‒	**+**	**+**	‒	‒	‒	**+**	‒	‒	‒	‒	‒	‒	**+**	**+**
	**FIIK**, HI2	1 (1.1)	‒	‒	‒	**+**	‒	‒	‒	‒	‒	‒	‒	‒	‒	‒	‒	**+**	**+**	**‒**
	HI2, Y	1 (1.1)	‒	‒	‒	**+**	‒	‒	‒	‒	‒	‒	‒	‒	‒	‒	‒	**+**	**+**	**+**
3	**FIIK**, HI2, Y	2 (2.2)	‒	‒	‒	**+**	‒	‒	‒	‒	‒	‒	‒	‒	‒	‒	‒	**+**	**+**	**‒**
	**FIIK**, R, HI2	1 (1.1)	**+**	‒	‒	‒	**+**	**+**	‒	‒	‒	‒	‒	‒	**+**	‒	‒	‒	**+**	**+**
	**FIIK**, R, I1	1 (1.1)	**+**	‒	‒	‒	**+**	**+**	‒	‒	‒	‒	‒	‒	**+**	‒	‒	‒	**+**	**+**
	**FIIK**, R, N	1 (1.1)	**‒**	‒	**+**	‒	**+**	‒	‒	‒	‒	‒	**+**	‒	‒	**+**	‒	‒	**+**	**‒**
	**FIIK**, FIB-M, HIB-M #	1 (1.1)	**+**	**‒**	**‒**	‒	**+**	**+**	‒	‒	‒	**+**	**‒**	**+**	‒	‒	‒	‒	**+**	**‒**
	**FIIK**, N, A/C	1 (1.1)	‒	‒	‒	**+**	‒	‒	‒	‒	‒	‒	‒	**+**	**+**	‒	‒	‒	**+**	**+**
	**FIIK**, FII, FIB	1 (1.1)	**+**	‒	‒	‒	**+**	**+**	**+**	‒	‒	**+**	‒	**+**	**+**	‒	‒	‒	**‒**	**+**
	FII, FIA, FIB	1 (1.1)	**+**	‒	‒	‒	**+**	**‒**	**+**	‒	‒	**‒**	‒	**+**	**+**	‒	‒	‒	**+**	**+**
4	**FIIK**, R, N, X1	1 (1.1)	‒	‒	‒	**+**	‒	‒	‒	‒	‒	‒	‒	‒	‒	‒	‒	‒	**+**	**+**
	**FIIK**, R, FIB-M, HIB-M *	1 (1.1)	**+**	‒	‒	‒	**+**	‒	**+**	**+**	‒	**+**	‒	**+**	**+**	**‒**	**+**	‒	**+**	**+**

+: detected, ‒: not detected, V: variable (detected in some isolates but not detected in others), FIIK is in bold, *strain NDM-2012 [14], # strain K24 [13]. Other SHV types (non-ESBLs) included: SHV-1 (8.7%), SHV-11 (39.8%), SHV-27 (3.3%), SHV-28 (11.8%), SHV-75 (1.1%), SHV-83 (5.4%) and SHV-144 (1.1%).

As shown in Table 2, isolates carrying the same replicon(s) exhibited variability with respect to the antibiotic resistance genes. In addition, a particular resistance gene was not confined to isolates harbouring a particular replicon or replicons combination, for e.g. CTX-M-15 was detected in *K*. *pneumoniae* isolates carrying various plasmid replicon types (e.g. FIIK, FIB-M, FIIK+R, FIIK+FIB-M and/or HIB-M, etc.).

DNA sequence analysis of FIIK replicons (n = 84; 90.3%) differentiated them into seven alleles. FIIK-2 was the most common allele detected from 45 (48.4%) isolates investigated in this study, followed by FIIK-7 (n = 23; 24.7%). Other less common FIIK alleles detected in this study were FIIK-4 (n = 1; 1.1%), FIIK-5 (n = 5; 5.4%), FIIK-8 (n = 5; 5.4%). Two novel alleles [FIIK-9 (n = 4; 4.3%) and FIIK-10 (n = 1; 1.1%)] were identified and the sequences had been deposited in the Plasmid MLST website.

Table 3 displays the detection rates of IncF plasmid replicon alleles in 93 *K*. *pneumoniae* isolates. The majority of the isolates (n = 81; 87%) had a single IncF replicon (FIIK). Replicon sequence typing of other IncF replicons (FIA, FIB and FII) identified different alleles [FIA-1 (n = 2; 2.2%), FIB-1 (n = 1; 1.1%), FIB-16 (n = 1; 1.1%), FII-1 (n = 1; 1.1%), FII-2 (n = 1; 1.1%) and FII-10 (n = 1; 1.1%)] in four isolates (Table 3). The isolates had two (2.2%) or three (2.2%) various IncF replicons, including FIIK plus one (either FII-10 or FIA-1) or two (FII-2 and FIB-1) IncF replicons or a combination of non-FIIK IncF replicons (FII-1, FIA-1 and FIB-16).

**Table 3:** The detection rates of IncF plasmid replicon alleles (FIIK, FII, FIA and FIB) in 93 *K*. *pneumoniae* isolates.

**FIIK alleles (n; %)**	**Co-detected IncF replicons (n; %)**	**FAB Formula**
FIIK-2 **(45; 48.4)**	None (44; 47.3)	K2: A-: B-
	FII-10 (1; 1.1)	K2, F10: A-: B-
FIIK-4 **(1; 1.1)**	None (1; 1.1)	K4: A-: B-
FIIK-5 **(5; 5.4)**	None (5; 5.4)	K5: A-: B-
FIIK-7 **(23; 24.7)**	None (21; 22.6)	K7: A-: B-
	FIA-1 (1; 1.1)	K7: A1: B-
	FII-2, FIB-1 (1; 1.1)	K7, F2: A-: B1
FIIK-8 **(5; 5.4)**	None (5; 5.4)	K8: A-: B-
FIIK-9 **(4; 4.3)**	None (4; 4.3)	K9: A-: B-
FIIK-10 **(1; 1.1)**	None (1; 1.1)	K10: A-: B-
Negative **(9; 9.7)**	FII-1, FIA-1, FIB-16 (1; 1.1)	F1: A1: B16
	None (8; 8.6)	None

n = number of isolates carrying each replicon,* Co-detected IncF replicons include FII, FIA and FIB. FAB (FII and/or FIIK: FIA: FIB) formula represents the allele type and number for each IncF replicon detected per isolate [10].

### Transmissibility of antibiotic resistance plasmids

Despite repeated attempts, conjugation experiment was successful for 27(62.8%) of 43 *K*. *pneumoniae* isolates which were selected as donors for conjugation based on their plasmid replicons (FIIK replicons alone or accompanied by different types of plasmid replicons) and ESBL genes (CTX-M-15, -3, -63 and SHV-12). Table 4 shows the characteristics of 43 donor *K*. *pneumoniae* isolates selected for conjugation experiments and the results of conjugation.

**Table 4:** Characteristics of 43 donor *K*. *pneumoniae* isolates selected for conjugation experiments and the results of conjugation.

*No*. *of replicons (n)*	*FIIK* r*eplicons*	*non-FIIK* r*eplicons*	*ESBL genes*	*No*. *of isolates*	*Conjugation result (n)*
*0 replicon*, *i*.*e*. *non-typable (4)*	‒	‒	CTX-M-15	4	N (2), **Y (2)**
*1 replicon (16)*	FIIK-8	‒	CTX-M-15	5	N (5)
	**FIIK-2**	‒	CTX-M-15	4	**Y (4)**
	**FIIK-7**	‒	CTX-M-15	2	**Y (2)**
	**FIIK-9**	‒	CTX-M-15	2	**Y (2)**
	‒	FIB-M	CTX-M-15	2	N (2)
	**‒ #**	N	CTX-M-3	1	**Y (1)**
*2 replicons (12)*	**FIIK-2**	R	CTX-M-15	3	**Y (3)**
	**FIIK-2**	FIB-M	CTX-M-15	1	**Y (1)**
	**FIIK-2**	HIB-M	CTX-M-15	1	**Y (1)**
	**FIIK-7**	R	CTX-M-15	1	**Y (1)**
	**FIIK-7**	N	CTX-M-15	1	**Y (1)**
	**FIIK-7**	FIB-M	CTX-M-15	1	**Y (1)**
	**FIIK-9**	I1	CTX-M-15	1	**Y (1)**
	**FIIK-9**	**R**	CTX-M-15	1	**Y (1)**
	FIIK-5	HI2	SHV-12	1	N (1)
	‒	HI2, Y	SHV-12	1	N (1)
*3 replicons (9)*	FIIK-4	R, N	CTX-M-63	1	N (1)
	**FIIK-2**	R, I1	CTX-M-15	1	**Y (1)**
	**FIIK-2**	FIB-M, HIB-M	CTX-M-15	1	**Y (1)**
	**FIIK-7**	FII-2, FIB-1	CTX-M-15	1	**Y (1)**
	‒	FII-1, FIA-1, FIB-16	CTX-M-15	1	N (1)
	**FIIK-2**	R, HI2	CTX-M-15	1	**Y (1)**
	FIIK-5	HI2,Y	SHV-12	2	N (2)
	FIIK-10	**N**, A/C	SHV-12	1	**Y (1)**
*4 replicons (2)*	FIIK-5	R, FIB-M, HIB-M	CTX-M-15	1*	N (1)
	FIIK-5	**R**, N, X1	SHV-12	1	**Y (1)**
***Total***				43	**Y (27)**

^#^ non-typable plasmid in a transconjugant (no replicon was detected).

**Y**: successful conjugation, N: failed conjugation, ‒: no replicon was detected, n: number of isolates

Bold underlined text: replicons transferred to the transconjugants,

*: NDM-2012 strain

As shown in Table 4, conjugation experiments were not successful for all donors carrying certain FIIK alleles [FIIK-4 (1.1%), FIIK-5 (5.4%) and FIIK-8 (5.4%)] in contrast to those having FIIK-2, -7 and -9 plasmids (100% success rate). The replicons detected in the transconjugants included FIIK (n = 22; 81.5%), N (n = 1; 3.7%) and R (n = 2; 7.4%). Three transconjugants [carrying CTX-M-15 (n = 2) and CTX-M-3 (n = 1)] were devoid of replicons (non-typable) in addition to one transconjugant which had both FIIK and R replicons.

A statistically significant association (p<0.05) between the presence of CTX-M-15 and FIIK was observed in this study as FIIK replicon was the dominant replicon detected in 22 of 24 CTX-M-15 harbouring transconjugants. On the other hand, SHV-12 gene was detected from two transconjugant plasmids carrying N and R replicon types, respectively.

Analysis of plasmid extracts from 43 *K*. *pneumoniae* donor isolates revealed the detection of 1–6 plasmids with size ranging from ~1.5–100 kb. A single plasmid was detected from most of the transconjugants (~100 kb in 18/27 transconjugants, ~60 kb and 90 kb in one transconjugant each). In seven transconjugants, the ~100 kb plasmid was accompanied by an additional of 1–2 plasmid(s) with smaller size [~5kb (n = 4), 5kb+3kb (n = 2) and 4kb (n = 1)]. Details of 43 *K*. *pneumoniae* isolates used as donors in the conjugation experiments, results of conjugation with plasmids size and number in the donors and transconjugants are shown in S2 Table.

### Characterization of the transconjugants

All the 27 transconjugants demonstrated β-lactamase activities using nitrocefin chromogenic detection method and were confirmed as ESBL producers by cefpodoxime combination disk method. Table 5 shows the comparison between *K*. *pneumoniae* donor isolates and their transconjugants (n = 27) with respect to non-susceptibility rates (%) and MICs of various antibiotics.

**Table 5:** Comparison between *K*. *pneumoniae* donor isolates and their transconjugants (n = 27) with respect to non-susceptibility rates (%) and MICs of various antibiotics.

Antibiotics	Non-susceptibility rates (%)		MICs (μg/ml)		
	D	T	D [range, MIC_50_, MIC_90_]	T [range, MIC_50_, MIC_90_]	*E*. *coli* strain J53 AzR (Recipient)
Ceftazidime	96.3	96.3	4-≥256, 24, ≥256	1.5-≥256, 16, 32	0.5
Cefotaxime	100	100	16-≥256, ≥256, ≥256	8-≥256, 96, ≥256	0.125
Aztreonam	100	100	16-≥256, 48, ≥256	8-≥256, 48, 64	0.094
Piperacillin-tazobactam	29.6	0	3-≥128, 8, ≥128	2–4, 2, 4	2
Ampicillin	100	100	ND	ND	S
Ceftriaxone	100	100	ND	ND	S
Cefuroxime	100	100	ND	ND	S
Cefoperazone	100	100	ND	ND	S
Amoxicillin-clavulanic acid	96.3	96.3	ND	ND	S
Ampicillin-sulbactam	100	100	ND	ND	S
Cefoxitin	7.4	0	ND	ND	S
Ciprofloxacin	70.4	0	0.125-≥32, 2, ≥32	0.016–0.75, 0.19, 0.75	0.016
Trimethoprim/sulfamethoxazole	96.3	88.9	0.38-≥32, ≥32, ≥32	0.125-≥32, ≥32, ≥32	0.125
Gentamicin	48.1	40.7	0.25–96, 0.75, 64	0.25–64, 0.5, 48	0.25
Amikacin	0	0	1–16, 3, 8	1–8, 1.5, 3	1

ND: MIC testing was not done, D: *K*. *pneumoniae* (Donors), T: Transconjugants (*E*. *coli* strain J53 AzR), S: susceptible

The transconjugants were resistant to multiple antibiotics including β-lactams, gentamicin and trimethoprim/sulfamethoxazole, but not ciprofloxacin. All the transconjugants demonstrated high resistance rates (96.3–100%) to β-lactam antibiotics except carbapenems and cefoxitin (0% for each). The transconjugants exhibited high resistance rates to β-lactam/β-lactamase inhibitor combinations (96.3% and 100% for amoxicillin-clavulanate and ampicillin-sulbactam, respectively); but were all susceptible to piperacillin-tazobactam (MIC 2–4 μg/ml) in contrast to some donors (29.6%) which were non-susceptible to this drug (MIC 48-≥128 μg/ml). None of the transconjugants demonstrated resistance to ciprofloxacin when compared to 70.4% of the donor *K*. *pneumoniae* isolates which were non-susceptible to ciprofloxacin (MIC 2-≥32 μg/ml).

All the transconjugants demonstrated significantly higher (p<0.05) MICs compared to those of the conjugation recipient strain (*E*. *coli* J53 AzR) for ceftazidime (3->500 folds), cefotaxime (64->2000 folds) and aztreonam (85->2000 folds).

Some of the transconjugants exhibited significantly higher (p<0.05) MICs for trimethoprim/sulfamethoxazole (>256 folds), ciprofloxacin (1.4->47 folds), gentamicin (1.5->256 folds) and amikacin (1.5–8 folds), compared to those of the recipient strain. However, other transconjugants demonstrated MICs equal to the corresponding values of the recipient strain. There was a slight increase in MICs for piperacillin-tazobactam (1.5–2 folds) noted in some transconjugants as compared to the conjugation recipient strain (*E*. *coli* J53 AzR).

MICs for the donor *K*. *pneumoniae* isolates were significantly higher (p<0.05) than those of the transconjugants for ceftazidime (1.5->16 folds), cefotaxime (1.3->4 folds), aztreonam (1.5->5 folds), piperacillin/tazobactam (1.5->64 folds), ciprofloxacin (2->2000 folds), gentamicin (1.3->64 folds) and amikacin (1.3–12 folds). However, the difference in trimethoprim/sulfamethoxazole MICs of donor *K*. *pneumoniae* isolates and their transconjugants was statistically non-significant (p>0.05).

Table 6 shows the detection of resistance determinants which were transferred from donors to transconjugants. Each transconjugant (n = 27) contained one ESBL gene including SHV-12 (n = 2) or CTX-M group-1 genes [CTX-M-15 (n = 24) and CTX-M-3 (n = 1)]. Other β-lactamase genes including TEM (n = 23), OXA-1 (n = 11) and SHV-11 (n = 4) were also detected in some transconjugants. DHA-1 AmpC β-lactamase gene and SHV β-lactamase genes from certain types (SHV-1, -27, -28, -75, -83 and -144) were not detected in any transconjugant. In addition to β-lactamase genes, co-transference of resistance genes against other antibiotic classes (aminoglycosides, fluoroquinolones and trimethoprim/sulfamethoxazole) was noted in most of the transconjugants (26 out of 27). These transconjugants carried an ESBL gene (either CTX-M-15 or SHV-12) accompanied by broad-spectrum β-lactamases (SHV-11, OXA-1 and TEM), PMQR genes (*aac(6′)-Ib-cr* and *qnrB*), aminoglycoside resistance gene (*aacC2* and *aac(6′)-Ib*) and trimethoprim/sulfamethoxazole resistance genes (*sul1* and *dfrA*). Some of the donor genes (SHV-11, *aacC2*, *aac(6ˊ)-Ib-cr*, *sul1* and *dfrA*) were not detected from their transconjugants (bold underlined text in Table 6).

**Table 6:** Resistance determinants detected in the donors and transconjugants in this study.

Antimicrobial category	Antibiotic resistance genes	Detection of resistance genes (n) in the donors (*K*. *pneumoniae*)	Detection of resistance genes (n) in the transconjugants (*E*. *coli* strain J53 AzR)
Beta-lactams	CTX-M-15*	24	24
	CTX-M-3*	1	1
	TEM	23	23
	OXA-1	11	11
	SHV-12*	2	2
	SHV-11	11	**4**
Aminoglycosides	*aacC2*	13	**11**
	*aac(6ˊ)-Ib*	1	1
Fluoroquinolones	*aac(6ˊ)-Ib-cr*	18	**17**
	*qnrB*	15	15
Trimethoprim/sulfamethoxazole	*sul1*	15	**14**
	*dfrA*	18	**17**

* ESBL genes, n: number of isolates. Bold underlined text: genes transferred from donors to some rather than all transconjugants, e.g. SHV-11 in 7 out of 11 donors was not transferred to their transconjugants.

The full list of 27 *K*. *pneumoniae* donor isolates and their transconjugants (*E*. *coli* strain J53 AzR) with their MICs and antibiotic resistance genes are shown in S3 Table. The successful transfer of resistance determinants (ESBL genes including CTX-M-15, CTX-M-3 and SHV-12, *aacC2* encoding gentamicin modifying enzyme, trimethoprim/sulfamethoxazole resistance genes including *sul1* and *dfrA*) caused non-susceptibility of each transconjugant to the corresponding antimicrobial category including β-lactams [ceftazidime (MICs 1.5-≥256 μg/ml), cefotaxime (MICs 8-≥256 μg/ml) and aztreonam (MICs 8-≥256 μg/ml)], gentamicin (MICs 16–64 μg/ml) and trimethoprim/sulfamethoxazole (MICs ≥32 μg/ml). Successful transfer of PMQR genes (*aac(6’)-Ib-cr* and/or *qnrB*) did not cause non-susceptibility of the transconjugants to ciprofloxacin (MIC 0.016–0.75 μg/ml) when compared to 70.4% of *K*. *pneumoniae* donor isolates which were non-susceptible to ciprofloxacin (MIC 2-≥32 μg/ml) due to the presence of chromosomal mutations in *gyrA* and/or *parC* regions with or without PMQR genes.

### Restriction analysis of transconjugant plasmids

**Fig 1:**
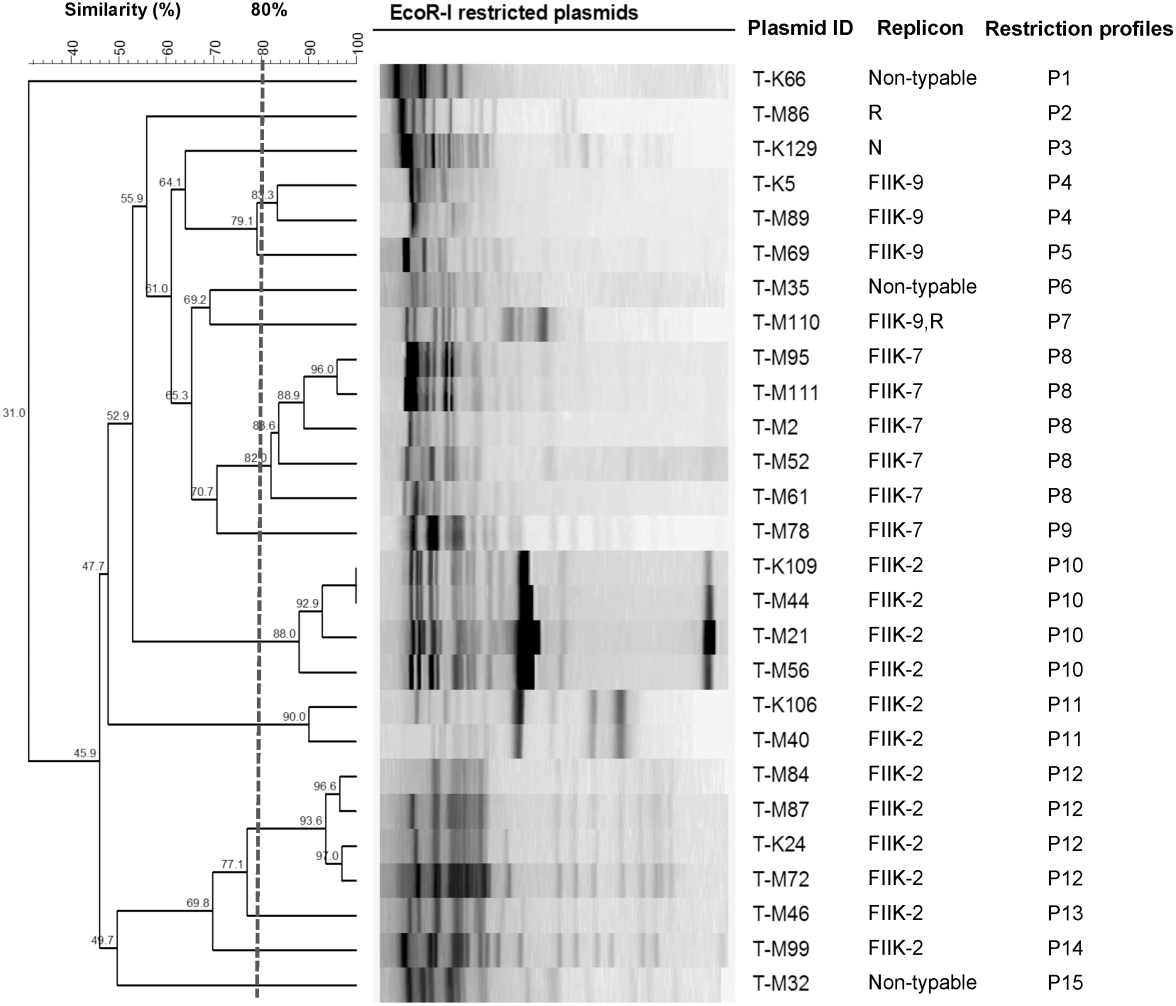
Dendrogram of EcoRI-digested plasmids from 27 transconjugants. 15 restriction profiles were identified (P1-P15). The dashed line represents the 80% similarity level used in cluster designation. Transconjugant plasmid ID, replicon and restriction profiles are shown.

A total of 15 different restriction profiles were obtained from the 27 *EcoRI*-digested transconjugant plasmids. Fig 1 shows the restriction profiles of the transconjugant plasmids.

A unique restriction profile (P1) was obtained from the ~60 kb transconjugant plasmid (T-K66) carrying CTX-M-3. Unique profiles (P2 and P3) were also obtained from the transconjugant plasmids (T-M86 and T-K129) carrying SHV-12 (~90 and 100 kb, respectively). A total of 12 restriction profiles (P4-P15) were obtained from the transconjugant plasmids carrying CTX-M-15 (~100 kb). One transconjugant plasmid with unique restriction profile was identified in each of seven clusters (P5, P6, P7, P9, P13, P14 and P15). The remaining five clusters (P4, P8, P10, P11 and P12) contained 2–5 transconjugant plasmids with highly similar (≥80%) restriction patterns.

## Discussion

In this study, CTX-M-15 was the prevalent ESBL gene detected among 91.3% of Malaysian multidrug-resistant *K*. *pneumoniae* isolates. The finding was in agreement with most recent studies in Asia and worldwide [22–24]. The dramatic shift of ESBL gene types from SHV to CTX-M has been noted globally as ESBL-SHV types are currently less common compared to CTX-M types [25, 26]. SHV gene was detected in 78.5% of the isolates; however, SHV-12 (6.5%) was the only ESBL-SHV gene detected in this study. SHV-5 has been previously identified as the most common ESBL gene in Malaysian *K*. *pneumoniae* isolated during a nosocomial outbreak in the pediatric oncology unit of University of Malaya Medical Center, Kuala Lumpur [27]. A subsequent study in this setting reported the emergence of SHV-12, a derivative from SHV-5 by acquisition of a single mutation (Leu35Gln) [28]. According to the latest SMART study, SHV-12 was the dominant ESBL-SHV gene in *K*. *pneumoniae* isolates from many parts of Asia-Pacific region including Malaysia, China, India, Philippines, Taiwan, Korea and Singapore [23].

This is the first report of SHV-27, -28, -75 and -83 types in Malaysian *K*. *pneumoniae* isolates. Some of these SHV types have been reported in other Asian countries, including SHV-28 which has been reported in *K*. *pneumoniae* isolates from China (GenBank accession no. AF538324), Korea [29] and India [30]. SHV-75 and SHV-27 have been documented in *K*. *pneumoniae* isolates from Thailand [24]. SHV-83 was detected for the first time in a *K*. *pneumoniae* isolate from Portugal [31], but has never been reported in South East Asia. The diversity in the SHV type of Malaysian *K*. *pneumoniae* isolates may occur through international spread by travelers or emerged via mutations in the parental SHV-1 or SHV-11 genes which are prevalent in the local isolates [32].

CTX-M-3 has been described as a common ESBL gene in some Asian countries including Korea, Taiwan and China [33, 34]. To the best of our knowledge, this is the first report of CTX-M-3 and CTX-M-63 (1.1% each) in Malaysian *K*. *pneumoniae* isolates. CTX-M-63 is an enzyme belongs to CTX-M group-8 which was first identified in a Japanese *K*. *pneumoniae* isolate in 2005 (GenBank accession no. AB205197), and later reported among isolates from other bacterial species including *Morganella morganii* (GenBank accession no. EU660216) and *Salmonella enterica* in Thailand [35].

OKP β-lactamases are chromosomally-encoded enzymes specific for *K*. *pneumoniae*, sharing the same ancestor origin with both SHV and LEN genes [36]. In this study, the detection of OKP gene in two isolates was by accident as the gene was amplified using SHV sequencing primers [21]. OKP β-lactamases are phylogenetically related to SHV; thus, the detection of OKP gene using SHV primers is possible as reported in a previous study [37]. In general, OKP gene is rarely reported in ESBL-producing *K*. *pneumoniae* isolates [38] and has not been reported before in South East Asia.

Except for a report of CMY detection in Malaysian *K*. *pneumoniae* isolates [39], limited information is available on AmpC genes in *Enterobacteriaceae* isolates in Malaysia. DHA-1 was identified recently in a Malaysian *K*. *pneumoniae* isolate in the SMART Asia-Pacific study [23]. The detection of DHA-1 gene in two isolates investigated in this study suggests the emergence of the AmpC gene among Malaysian *K*. *pneumoniae* isolates. Additionally, regional variations in the distribution of AmpC genes has been reported in *Enterobacteriaceae* isolates from the Asia-Pacific region according to the latest SMART study [23]. DHA-1 gene was more common in *Enterobacteriaceae* isolates from Philippines and Singapore compared to CMY gene which was dominant in *Enterobacteriaceae* isolates from Taiwan, India, South Korea and Vietnam [23].

A big proportion (74%) of the multidrug resistant *K*. *pneumoniae* isolates investigated in this study were non-susceptible to gentamicin. Resistance to gentamicin is common in ESBL-producing *K*. *pneumoniae* isolates, as reported in several parts of the world including China [3] and Taiwan [40]. In this study, only a few isolates (5.4%) were non-susceptible to amikacin. This finding concurs with recent surveillance studies of *K*. *pneumoniae* isolates from the Asia-Pacific region which indicted that less than 10% of the isolates were non-susceptible to amikacin [1,41].

The association between non-susceptibility to gentamicin and the presence of *aacC2* gene encoding the gentamicin modifying enzyme AAC(3)-II have been documented [42,43]. In agreement with these reports, *aacC2* was detected in most gentamicin non-susceptible isolates in this study. Additionally, one of the gentamicin-resistant isolates harboured *aadB* gene which is known for conferring resistance to gentamicin [44]. *aadB* gene has been reported recently in *K*. *pneumoniae* isolates from Malaysia [22,45], in contrast to *aacC2* gene which is reported for the first time in Malaysia. Both *aacC2* and *aadB* genes have been reported in gentamicin-resistant *K*. *pneumoniae* isolates from China, with higher prevalence of the former (60%) than the latter (3.6%) [3]. Similar observation was noted in this study as *aacC2* gene (67.7%) was more common than *aadB* gene (1.1%). *aacC1*, which confers resistance to gentamicin, has been detected at a very low rate (2.7%) among aminoglycoside-resistant *K*. *pneumoniae* isolates in a Chinese study [3]; however, it was not detected in this study.

High-level resistance to multiple aminoglycosides is common in *K*. *pneumoniae* due to the spread of 16S rRNA methylases in multidrug resistant bacteria worldwide [3]. Except for strain NDM-2012 carrying *armA* gene [14], none of the isolates understudied were positive for 16S rRNA methylases (*armA* and *rmtB*) or phosphotransferase gene (*aphA6*), two genes associated with amikacin resistance [43].

Trimethoprim/sulfamethoxazole resistance is commonly observed in ESBL-producing *K*. *pneumoniae* isolates [3]. High resistance rate against trimethoprim/sulfamethoxazole (90.3%) as noted in this study, was probably attributed to the presence of *sul1* and/or *dfrA* genes. In fact, both *sul1* (encoding dihydropteroate synthases) and *dfr* (encoding dihydrofolate reductase) have been reported as causes of trimethoprim/sulfamethoxazole resistance in Gram-negative bacteria [46].

There is limited data on the plasmids of multidrug resistant *K*. *pneumoniae* isolates in Malaysia. The detection of 1–6 plasmids (~1.5–100 kb) in our isolates reflects the complexity of multidrug resistant isolates, in agreement with a previous study which described significantly higher number of plasmids in multidrug resistant *K*. *pneumoniae* isolates [47]. The existence of several plasmids within the same isolate may increase the possibility of genetic reassortment and recombination events and contribute to the plasmid diversity by recruiting new resistance genes into the plasmid scaffold [48].

In this study, 1–4 of 14 plasmid replicons types were detected from majority (95.7%) of the isolates. The finding suggests the existence of highly diverse plasmids carried by the Malaysian multidrug resistant *K*. *pneumoniae* isolates. The FIIK replicon was the dominant replicon type (90.3%) which was identified as a single replicon in more than half (53.8%) of the 93 isolates investigated in this study. *K*. *pneumoniae* isolates carrying FIIK replicon has been reported to have great ability to diffuse and persist in time [49,50], mainly because the bacteria are equipped with both virulence and antibiotic resistance determinants on the FIIK plasmids [10].

The detection of a few isolates (4.3%) carrying IncF replicons (FIIK, FIA, FIB and/or FII) is in line with previous studies which reported the detection of multi-IncF-replicon plasmids in *K*. *pneumoniae* and other species from *Enterobacteriaceae* [8,47]. IncF plasmids are notorious for their ability to evolve rapidly in order to adapt to the host environment; thus, mutations and recombination are common in these plasmids [10,51]. Sequence analysis revealed diversity in the IncF replicons identified in this study. FIIK replicons were differentiated into seven alleles based on sequence variation, of which, two were considered novel (FIIK-9 and -10). In addition, various alleles from FIA, FIB and FII replicon types were identified in this study.

There is a paucity of data on FIIK alleles around the world due to the limited publications available in this field [10,11]. Most studies investigating plasmid replicon types in *K*. *pneumoniae* used the old version of PCR-based plasmid replicon typing (PBRT) method developed in 2005 [17], which utilized primers specific for the detection of 18 replicons including IncF replicons (FIA, FIB, FIC, FII and F), but not for FIIK replicon [17]. The new PBRT scheme updated in 2010 has included new primers for the detection of IncF plasmids specific for *Salmonella*, *Klebsiella* and *Yersinia spp*. (FIIS, FIIK and FIIY, respectively) [10]. Hence, the real prevalence of FIIK might not been updated in many parts of the world [47,52]. The only database available for comparative analysis of FIIK alleles is provided by the Plasmid MLST (pMLST) website (http://pubmlst.org/plasmid/).

The finding of FIIK-2 as the most common allele (48.4%) in this study is in agreement with the pMLST records which showed the predominance of FIIK-2 allele in 35.7% of FIIK-carrying isolates. The second most common FIIK allele in this study, FIIK-7 (24.7%), has also been detected in multidrug resistance plasmids in *K*. *pneumoniae* isolates from Czech Republic [11]. Other IncF replicons [FII (3.2%), FIA (2.2%) and FIB (2.2%)] identified in this study were less common compared to FIIK replicon. FIA-1 and FII-2 alleles have been reported in *K*. *pneumoniae* isolates from South Korea [51] and Italy [10], respectively. Other alleles (FII-1, FII-10, FIB-1 and FIB-16) have not been reported in *K*. *pneumoniae*; however, most of them (FII-1, FIB-1 and FIB-16) were common in *E*. *coli* isolates from several geographical regions including Japan, Tunisia and Spain (http://pubmlst.org/plasmid/).

The second most common replicon identified in this study was the R replicon which was detected in 20.4% of the isolates harbouring FIIK replicon. Both FIIK and R replicons have been described previously to have an association with CTX-M-15 in Spanish *K*. *pneumoniae* isolates [49]. The R replicon has also been identified on a resistance plasmid associated with the spread of KPC gene amongst Canadian *K*. *pneumoniae* isolates [53] and on resistance plasmids bearing CTX-M-15, *qnr* and/or *aac(6′)-Ib-cr* in Spanish *K*. *pneumoniae* isolates [54]. These reports suggest the contribution of R replicon in the dissemination of resistance genes.

The remaining replicons detected in this study (FIB-M, HIB-M, N, HI2, Y, I1, A/C, X1 and K) were less common (1.1–7.6%) among the isolates. This was expected as some plasmid replicon families have a narrower distribution in certain geographical regions [7,9]. Strain NDM-2012 (carrying both NDM-1 and OXA-232 genes), the only carbapenem-resistant isolate investigated in this study, harboured four plasmid replicons i.e. FIIK, R, FIB-M and HIB-M, of which, the latter two are novel replicons reported from the plasmid of a Moroccan *K*. *pneumoniae* isolate carrying NDM-1, CTX-M-15 and *qnrB1* genes [55]. FIB-M and HIB-M were not limited to strain NDM-2012 as they were also detected in several isolates investigated in this study. Plasmids can be exchanged among different isolates. Additionally, the resistance genes can be acquired or lost from a plasmid scaffold; thus, a particular resistance gene may not be linked exclusively to plasmids from a specific replicon family [8]. This may explain our observation of isolates carrying the same replicon(s) but exhibiting variability with respect to the antibiotic resistance genes.

Conjugation is the most common mechanism of horizontal dissemination of resistance plasmids with much higher success rates in nature than under laboratory conditions [6,56]. In this study, conjugative transfer of ESBL plasmids to *E*. *coli* strain J53 AzR recipient strain was only successful in 27 (62.8%) of 43 *K*. *pneumoniae* donor isolates. Approximately same rate (61%) of conjugation has been documented for multidrug resistant *K*. *pneumoniae* isolates investigated in a Czech study [11]. The success rate of conjugation could be affected by the selection of the recipient strain. Higher conjugative transfer rates of ESBL plasmids have been observed from *K*. *pneumoniae* to *E*. *coli* (47%) than to *Salmonella* (20%) recipient strains [11]. In this study, conjugation was only attempted using *E*. *coli* strain J53 AzR (kindly provided by Dr. Jacoby G.A.), a recipient strain widely used for conjugation experiments [3].

The replicative and transferability properties of plasmids are related to their incompatibility groups and a relationship may exist between FIIK allele and conjugative efficiency of the plasmids [6]. IncF plasmids are characterized by extensive mutations, insertions, deletions and recombination events which may affect the *tra* genes encoding transferase proteins for mating aggregation and DNA movement into the recipient cell and, consequently, plasmids conjugative efficiency [57, 58]. All plasmids carrying FIIK-4 (1.1%), FIIK-5 (5.4%) and FIIK-8 (5.4%) were not successfully transferred to the *E*. *coli* recipient strain. The loss or mutations in *tra* genes in the plasmids carrying certain FIIK alleles such as FIIK-4, -5 and-8 in this study might have resulted in less efficient donors for conjugation [58]. Hence, FIIK-4, FIIK-5 and FIIK-8 replicons were found less common (1.1%, 5.4% and 5.4%, respectively) as compared to FIIK-2 and FIIK-7 replicons (48.4% and 24.7%, respectively) in the isolates understudied.

It has been reported that conjugative plasmids are bigger in size compared to the non-conjugative counterparts [59]. Several authors have reported the recovery of conjugative resistance plasmids of ≥30 kb from multidrug resistant *K*. *pneumoniae* isolates [11,48,54]. However, resistance genes can be carried by small plasmids (<10 kb), also known as mobilizable resistance plasmids, which can be disseminated to a new host with the help of the conjugative plasmids [59, 60]. In agreement with these reports, this study showed the transfer of a big plasmid (~60–100 kb) by conjugation from 27 donor *K*. *pneumoniae* isolates to the *E*. *coli* recipient strain. The big plasmid was accompanied by 1–2 small (~3–5 kb) plasmids in seven transconjugants. The co-transfer of multiple plasmids from donors *K*. *pneumoniae* isolates to *E*. *coli* recipient strain has been reported previously [19]. The small plasmids may be mobilizable resistance plasmids or helper plasmids which provide mobilization proteins essential for conjugation [7].

In this study, SHV-12 ESBL gene was detected from two transconjugant plasmids carrying N and R replicon types, respectively. Both replicons have been previously documented in plasmids carrying SHV-12 in *K*. *pneumoniae* isolates [61,62]; however, SHV-12 was not confined to plasmids with a particular replicon type due to the ability of this gene to move among different plasmid scaffolds [8,63]. The association between the presence of CTX-M-15 and FIIK plasmid replicon on transconjugant plasmids was noted in this study, in agreement with previous reports [10,11,48]. Three transconjugant plasmids carrying CTX-M-15 (n = 2) and CTX-M-3 (n = 1) were devoid from any replicons. Non-typable plasmids (lacking replicons) have been previously reported in antibiotic resistant *K*. *pneumoniae* [47,64] and other members of *Enterobacteriaceae* [8].

Full sequencing of resistance plasmids in *K*. *pneumoniae* [65,66] and other bacterial species including *E*. *coli* [58,67] showed the structural linkage of various antibiotic resistance genes which were clustered in a specific region within the plasmid known as multi-resistance region. In this study, ESBL genes (CTX-M-15 or SHV-12) amplified from the transconjugant plasmids were accompanied by various collections of other resistance genes including those encoding broad-spectrum β-lactamases (SHV-11, OXA-1 and TEM), PMQR genes (*aac(6′)-Ib-cr* and *qnrB*), aminoglycoside resistance gene (*aacC2* and *aac(6′)-Ib*) and trimethoprim/sulfamethoxazole resistance genes (*sul1* and *dfrA*). The detection of multiple antibiotic resistance genes from the same plasmid suggests the possibility of having multi-resistance regions carried in the plasmids of the Malaysian *K*. *pneumoniae* isolates. Full plasmid sequencing approach is required to prove this hypothesis.

Some of the genes detected in the donors (*aacC2*, *aac(6ˊ)-Ib-cr*, DHA-1, *sul1* and *dfrA*) were not detected from the transconjugants. This may be attributed to their locations on a different plasmid other than the ESBL plasmid transferred during the conjugation experiment [19]. Furthermore, chromosomal location of these genes may be suspected as recent reports have confirmed the location of some plasmid-mediated antibiotic resistance genes such as *aac(6′)-Ib-cr* and *armA* on *K*. *pneumoniae* chromosome [54,68].

In general, MICs of β-lactams, β-lactam/β-lactamase inhibitors, aminoglycosides, and fluoroquinolones of the transconjugants were significantly lower than their donor *K*. *pneumoniae* isolates. This finding suggests the presence of other resistance mechanisms in the donors, for instance, chromosomal-mediated resistance genes, reduced intracellular drug accumulation due to active efflux pump and/or porin loss [69,70].

All the transconjugants carrying ESBL genes [SHV-12 (n = 2), CTX-M-3 (n = 1) and CTX-M-15 (n = 24)] demonstrated high resistance rates (96.3–100%) to β-lactam antibiotics except carbapenems and cefoxitin (0% for each). The transconjugants exhibited high resistance rates to β-lactam/β-lactamase inhibitor combinations (96.3% and 100% for amoxicillin-clavulanate and ampicillin-sulbactam, respectively); but were all susceptible to piperacillin-tazobactam. In fact, the ability of β-lactam/β-lactamase inhibitor combination to inactivate β-lactamases is dependent on the total quantity of the enzyme that needs to be inhibited; thus, β-lactamases hyperproduction or the concomitant presence of multiple β-lactamases may reduce the bacterial susceptibility to these combinations [71–73]. It is possible that lower β-lactamases net amount was produced by the transconjugants due to the absence of the chromosomal non-ESBL SHV enzymes (SHV-1, -28, -27, -75, -83, -144, -11) from these transconjugants; thus affecting their susceptibility levels to piperacillin-tazobactam [73,74]. None of the transconjugants demonstrated resistance to ciprofloxacin when compared to some of the donor *K*. *pneumoniae* isolates which were resistant to ciprofloxacin due to the presence of chromosomal *gyrA* and/or *parC* mutations [12]. All gentamicin-resistant transconjugants were positive for *aacC2* gene, confirming the role of this gene in conferring resistance to gentamicin [42]. Trimethoprim/sulfamethoxazole resistance was detected in transconjugants carrying *sul1* and/or *dfrA* genes, confirming the role of these genes in conferring resistance to trimethoprim/sulfamethoxazole [46].

Restriction analysis of plasmids extracted from 27 transconjugants in this study revealed high diversity of these plasmids (15 profiles) even amongst plasmids of the same replicon type. Genetic events such as insertions, deletions, reassortment and recombination may have happened during plasmid evolution contributing to the observed plasmid diversity [58,64]. The finding of diverse plasmids carrying multiple resistance genes in the Malaysian *K*. *pneumoniae* isolates was on the contrary to the findings of a recent Chinese study whereby, a single epidemic plasmid was implicated in the spread of CTX-M-15 gene in the *K*. *pneumoniae* isolates [19]. In that study, conjugative transfer of a 90 kb IncFII plasmid was documented from *K*. *pneumoniae* isolates collected from different hospitals in southern China. These plasmids were highly related as indicated by restriction analysis of transconjugant plasmids carrying CTX-M-15 gene alone in contrast to the parental *K*. *pneumoniae* isolates which harboured multiple resistance genes such as DHA-1, *qnrB*, *qnrS*, *aacC2*, and *aac(6ˊ)-Ib* [19]. These findings indicate that the plasmids circulating among *K*. *pneumoniae* isolates from different geographical locations may vary in the type and distribution of resistance genes [6].

In conclusion, this is the first study describing the characterization of plasmids in Malaysian multidrug resistant *K*. *pneumoniae*. The results of this study suggest that highly diverse plasmids with multiple antibiotic resistance determinants are spread among the Malaysian isolates. The location of resistance genes on conjugative plasmids and the ability for co-transference *en bloc* is an alarming finding as their dissemination may increase multidrug resistance rates among the Malaysian *K*. *pneumoniae* isolates unless more strict infection control measures and antibiotic stewardship programs are adopted to limit the spread of the multidrug resistant bacteria.

## Supporting Information

S1 Table Plasmid replicons and antibiotic resistance genes detected in 93 *K*. *pneumoniae* isolates. (XLSX)

S2 Table Details of 43 *K*. *pneumoniae* isolates used as donors in the conjugation experiments. The isolates were arranged according to the results of conjugation experiments. Plasmids size and number are shown for the donors and transconjugants. FIIK replicons in the transconjugant plasmids were detected by PCR and confirmed by sequence analysis of 8 representative amplicons (indicated by asterisk).
(XLSX)

S3 Table MICs (μg/ml) of various antibiotics and resistance determinants detected in *K*. *pneumoniae* donor isolates and the transconjugants. (XLSX)
